# Behavioral Factors Associated with COVID-19 Risk: A Cross-Sectional Survey in Japan

**DOI:** 10.3390/ijerph182212184

**Published:** 2021-11-20

**Authors:** Sae Ochi, Mirai So, Sora Hashimoto, Kenzo Denda, Yoichi Sekizawa

**Affiliations:** 1 Department of Laboratory Medicine, The Jikei University School of Medicine, Tokyo 105-0003, Japan; 2 Department of Psychiatry, Tokyo Dental College, Tokyo 101-0061, Japan; somirai@tdc.ac.jp; 3 United Health Communication Co., Ltd., Tokyo 103-0006, Japan; hashimoto@uhc.jp; 4 Hiramatsu Memorial Hospital, Sapporo 064-8536, Japan; denda_kenzo_0330@yahoo.co.jp; 5 Research Department, Research Institute of Economy, Trade and Industry, Tokyo 100-8901, Japan; sekizawa-yoichi@rieti.go.jp

**Keywords:** COVID-19, SARS-CoV-2, behavioral change, remote work, exercise

## Abstract

Background: Behaviors to avoid infection are key to minimizing casualties of the COVID-19 pandemic, as well as to avoid excessive interventions that are less effective. This study aims to identify behavioral patterns associated with SARS-CoV-2 infection in the real world. Methods: A questionnaire-based cross-sectional study was conducted targeting a research panel of NTTCom Online Marketing Solutions Corporation or its affiliates. Data were extracted so that their demographic composition ratios matched the population estimates. Individuals who answered with consistency to have been diagnosed with SARS-CoV-2 at a medical facility were categorized into a SARS-CoV-2 group. Differences in lifestyles were compared using multiple regression and inverse probability weighing. Results: In total 13,277 participants were included, of whom 44 (0.33%) were categorized as the SARS-CoV-2 group. Diagnosis of SARS-CoV-2 was negatively correlated with crowd avoidance, mask wearing, and hand-washing behavior. On the contrary, the diagnosis was positively correlated with some behaviors that appear to be preventive actions against the infection, such as changing clothes frequently, sanitizing belongings, and remote working. Conclusions: It is important to conduct evidence-based intervention on people’s behaviors and to avoid excessive interventions that are less effective, so that people can minimize the indirect harm, such as exhaustion and economic loss.

## 1. Introduction

COVID-19, a syndrome caused by SARS-CoV-2, has dramatically changed the lifestyles of people all over the world. Although a sense of normalcy is beginning to return in some countries due to vaccine development and introduction, there are cases of ‘breakthrough infection’ among those who are fully vaccinated [1]. Therefore, it is expected to take some time before the infection becomes under control. Therefore, traditional public health measures, including infection-avoidance behavior of each individual, are still highly important to minimize casualties of the infection [2].

Even so, many people feel fatigued by large-scale restrictions on their movements, including lockdowns and curfews. Excessive regulations can greatly and negatively affect people’s physical and mental health [3], as well as economic status [4,5]. A systematic review suggested deterioration of mental health might be a global health problem [6]. There is also a concern about increase in domestic violence and substance abuse [7]. Particularly in Japan, an increase in the suicide rate among women in Japan has been reported [8], which is attributed to anxiety about their children’s health, increase in domestic violence, and high frequency of lay-offs. Not only population health, but also the healthcare system itself, might be affected by austerity measures [9]. To minimize such indirect negative impacts of the pandemic on public health, prevention measures should not only be effective, but also be lean so that people are not exhausted by the long-term excessive restrictions of their behaviors.

SARS-CoV-2 has only two simple transmission routes: via inhalation of droplets scattered by an infected person’s coughing or talking, or via touching one’s eyes, nose, or mouth with a contaminated hand [10]. General measures for individuals include mask use, hand washing, ventilation of a space, and distancing from other people [11]. In addition to these, there are often governmental interventions such as lockdowns, curfews, and induction of remote works. All of these measures are effective in many cases, but the effectiveness of each measure differs by region and culture. For example, “social distancing” can be a priority in Europe and the U.S., where there is a custom of hugging and handshaking. This measure may not necessarily be a priority in Asian countries where people bow when greeting each other. On the other hand, hand washing might be more important in many Asian countries where there is a custom of eating with one’s hands [12]. Thus, it is necessary to prioritize behavioral interventions based on epidemiological evidence to reduce the infection risk at a regional level.

In this study, behavioral patterns associated with a COVID-19 diagnosis were analyzed based on the results of a large-scale questionnaire survey in Japan. By identifying effective preventive measures in the real world, this research will contribute to prioritizing protective measures that are both effective and sustainable.

## 2. Materials and Methods

### 2.1. Patient and Public Involvement

Data were collected as part of the research project, “Basic research for exploring the ideal medical intervention after the advent of the new coronavirus”, of the Research Institute of Economy, Trade, and Industry (RIETI). The online survey was called, “the 2020 Continuing survey on mental and physical health during the COVID-19 pandemic” (hereinafter RIETI questionnaire survey), and NTTCom Online Marketing Solutions Corporation was commissioned to conduct it. The data used in our study were microdata of the first survey conducted during the period 27 October–6 November 2020. The content of the questions is presented in Supplementary Table S1.

### 2.2. Data Collection

The surveyed subjects were men and women Japan-wide aged 18–74 years and were members of a research panel of NTTCom Online Marketing Solutions Corporation or its affiliates. They were extracted so that their demographic composition ratios of sex, age, and distribution of prefectures matched the population estimates of the Statistics Bureau of Japan (final estimates, May 2020). The final number of respondents was targeted to be approximately 15,000.

Data were excluded when: individuals provided non-existent zip codes; zip codes did not match the given prefectures; there were extreme outlying values for height and weight (200 cm or more for height, and less than 35 kg or 100 kg or more for weight, which is abnormal in Japan); age differed by 2 years or more from that previously given in the survey company’s registration; or response time was very short (less than 5 min) or very long (10 h or more). The remaining individuals were recognized as valid respondents.

### 2.3. Statistical Analysis

#### 2.3.1. Outcome Variables

The outcome index used in this study was the SARS-CoV-2 diagnosis status. If an individual chose the answer, “I have been diagnosed as SARS-CoV-2 infection at a medical facility and am currently under treatment” or “I was diagnosed as SARS-CoV-2 infection at a medical facility and have already recovered”, then he/she was categorized into a SARS-CoV-2 group and the presence of diagnosis was used as the primary outcome variable. This study established that the subjects “experienced SARS-CoV-2 infection” only if they were diagnosed with it at a medical facility.

The questionnaire was conducted 3 times: in October 2020, January 2021, and May 2021. If there was a discrepancy between the answer about SARS-CoV-2 diagnosis (e.g., a participant answered,”I was diagnosed as SARS-CoV-2 infection” in the first questionnaire and “Not diagnosed” in the second one), the data were omitted.

#### 2.3.2. Explanatory Variables

In addition to the SARS-CoV-2 diagnosis status, this survey asked the questions below regarding underlying disease and behavior. The detail of each question is shown in Table S1.

- Pre-existing diseases;
- Behaviors to avoid contracting SARS-CoV-2;
- Average days and hours of exercise in a week;
- Main exercise type;
- Change in the amount of exercise compared with the same time last year;
- Frequency of going out;
- Frequency of working from home in the past one month.

#### 2.3.3. Comparison of the Two Groups

To compare the two groups, Wilcoxon’s rank sum test and chi-square test were used for continuous variables and categorical variables, respectively.

#### 2.3.4. Multivariate Analysis

Logistic regression analysis was performed to assess the relationship between outcome and explanatory variables after adjustment for age, sex, and body mass index (BMI). To minimize the effects of outliers, a robust method was applied for the following regression tests.

The proportion of patients diagnosed as SARS-CoV-2 infection was very low. Thus, this study also used inverse probability weighting (IPW) to estimate the average treatment effect (ATE) of each item on SARS-CoV-2 infection and on the risk of infection symptom occurrence. In IPW, a propensity score is used to weigh each observed value in the sample. Two types of expected values are then estimated: the expected value of the outcome if the treatment is used for the overall sample (in this analysis, if individuals had travelled) and the expected value of the outcome if the treatment is not used. The ATE is estimated from the difference between these values.

Specifically, the inverse of the estimated propensity score (1/∂) is used for weighting. The inverse of a propensity score increases as the propensity score decreases. Therefore, a smaller weight is given to an observed value with a larger propensity score in the treated group, and a larger weight is given to an observed value with a larger propensity score in the control group. In other words, calculation is done with more weighting for an observed value that is rarer or accounts for a smaller proportion of the sample for each of the treated group and control group.

The statistical analyses were carried out using Stata/SE 16.0 (StataCorp LLC, College Station, TX, USA).

#### 2.3.5. Sensitivity Analysis

Sensitivity analysis was conducted targeting the participants who answered to have been diagnosed as SARS-CoV-2 in the survey in May 2021 only.

#### 2.3.6. Ethical Considerations

All individuals who participated in this study consented to their participation. This study was conducted with the approval of the ethics committee of Hiramatsu Memorial Hospital affiliated with Specified Jisoukai Medical Corporation (ID of approval: 20200925).

## 3. Results

### 3.1. Background of the Responders

There was a total of 19,340 respondents during the survey period, of whom 6063 were excluded because the reliability of their responses could not be fully ensured. As a result, the number of analyzed subjects was 13,277 (6582 males and 6739 females), of whom 44 (0.33%) were validated as the SARS-CoV-2 group and 13,277 were in the control group.

Table 1 shows the background factors of the two groups. The SARS-CoV-2 group included a higher proportion of younger people compared with the control group. The proportion of coexistence of heart disease was also higher in the SARS-CoV-2 group (11.4% in the SARS-CoV-2 group vs. 2.3% in the control group).

For lifestyle factors, a lower proportion of the SARS-CoV-2 group avoided crowded places (65.9% in the SARS-CoV-2 group vs. 87.1% in the control group), wore a mask (84.1% vs. 97.3%), and washed hands (77.3% vs. 96.5%). On the contrary, a higher proportion of the SARS-CoV-2 group changed clothes frequently (50.0% vs. 21.2%) and disinfected their belongings (54.5% vs. 28.2%). The proportion of those who worked from home largely all of the time was higher among the SARS-CoV-2 group than the control group (68.2% vs. 60.9%).

### 3.2. Multivariate Logistic Regression Analysis

**Figure 1:**
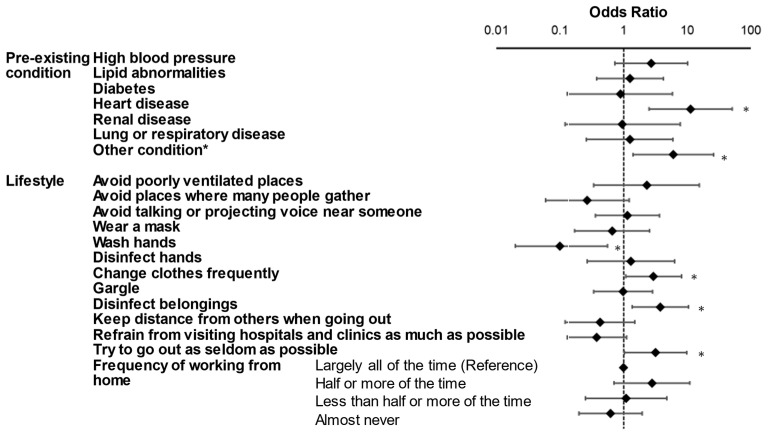
Odds ratio of COVID-19 infection by multivariate logistic regression for pre-existing conditions and lifestyle factors. * *p* < 0.05.

Multivariate logistic regression analysis was conducted (Table 2, left column and Figure 1).

Age was negatively correlated with diagnosis (odds ratio (OR) 0.94 per year, 95% confidence interval (CI) 0.91–0.98, *p* < 0.01 in multiple regression), while coexistence of heart disease (OR 11.33, 95%CI 2.50 to 51.25, *p* < 0.01) and other conditions (OR 6.03, 95%CI 1.41 to 25.77, *p* = 0.02) were positively associated with SARS-CoV-2 infection. As for lifestyle factors, washing hands (OR 0.10, 95%CI 0.02 to 0.56, *p* = 0.01) was negatively associated with infection. Interestingly, the diagnosis was significantly and positively correlated with changing clothes frequently (OR 2.96, 95%CI 1.08 to 8.15, *p* = 0.04), sanitizing belongings (OR 3.78, 95%CI 1.37 to 10.44, *p* = 0.01), and avoiding outings (OR 3.20, 95%CI 1.03 to 9.88, *p* = 0.04).

### 3.3. Analysis Using Inverse Probability Weighting Method

As sample size of the SARS-CoV-2 group was small, an IPW analysis was also conducted, controlling for background factors that showed significant differences in multiple regression, that is, age and coexistence of heart disease (Table 2, right column).

Habit of crowd avoidance (ATE −62.2, 95%CI −115.6 to −8.7, *p* = 0.02) and hand washing (ATE −84.8, 95%CI −155.8 to −13.7, *p* = 0.02) were negatively correlated with SARS-CoV-2 infection. In contrast, habits of changing clothes frequently (ATE 274.4, 95%CI 113.2 to 435.6, *p* < 0.01) and sanitizing their belongings (ATE 100.0, 95%CI 55.9 to 144.1, *p* < 0.01) were positively associated with the infection, which was consistent with the results of the logistic regression. In addition, no or rare remote work (ATE −77.1, 95%CI −143.4 to −0.2, *p* = 0.02) were negatively correlated with infection compared with almost daily remote work, which was contrary to the common thinking that remote working is effective in infection prevention.

### 3.4. Sensitivity Analysis

For sensitivity analysis, the same analysis in Table 2 was conducted among those who responded as being diagnosed as SARS-CoV-2 in the third survey (thus, their answers were not fully validated). In total, 110 were included in the SARS-CoV-2 group and 16,365 in the control group. In this analysis, habit of changing clothes frequently and sanitizing their belongings were consistently and positively associated with SARS-CoV-2 infection in IPW analysis (Table S2).

## 4. Discussion

This study is the first Japan-wide study that analyzed behavioral factors associating with SARS-CoV-2 infection in detail. It reconfirmed the effectiveness of mask wearing and hand washing in risk reduction. At least for infection prevention, the study did not show effectiveness of excessive behavior, such as frequent changing of clothes and extreme reduction of outings.

The most notable finding is that remote working and restrictions on outings did not always reduce the risk of COVID-19. Instead, these actions even appeared to increase the risk of the infection. This is contrary to previous analysis that showed effectiveness of lockdown [13,14]. There could be several reasons for this result. One possibility is that remote working and restrictions on outings gave a false sense of security and individuals began to neglect hand washing and mask wearing. A study of one Massachusetts city examined the genetic material of SARS-CoV-2 attached to surfaces to investigate the virus in the environment. PCR was positive in approximately 8% of the samples taken from environmental surfaces, and a particularly high level of virus was attached to the surfaces of trash cans [15]. Thus, even if outings are restricted, individuals cannot completely avoid their contact with environmental surfaces. Therefore, the infection risk could increase, especially if there is inadequate hand washing. Another possibility is that, even if individuals work remotely, they could be engaging in other high-risk behavior such as eating out with multiple individuals.

Our research also revealed that frequent changing of clothes and sanitizing belongings were significantly and positively associated with the infection risk. The result, however, does not mean that wearing and removing clothes increase the infection risk. It instead suggests that individuals who engage in such behavior might have limited knowledge of infection—they could be implementing ineffective preventive measures while neglecting the practice of highly effective ones. It is also possible that frequent changing of clothes could be a sign of mental disorder triggered by anxiety of infection, which has been reported to increase the COVID-19 risk [16].

In general, a moderate level of exercise is necessary for reduction of health risk. Our study showed walking may have a preventive effect of infection. However, our research also indicated that a high infection risk was correlated with 4 or more days of exercise, 30 min^−1^ h duration, and running was associated with a higher proportion of infection. The result suggests that individuals might have increased their contact with the virus by going out to exercise or by the use of a gym. Even so, a moderate level of exercise decreased the risk of severe illness from infection. It also has a preventive effect on other conditions (including diabetes, obesity, and hypertension) which increases the risk of severe illness from infection. Therefore, individuals should not unnecessarily avoid exercising.

The findings of this study strongly suggest that we may need a strategy other than legislation to change behaviors of populations. Epistemic communities, defined as “a network of professionals with recognized expertise and competence in a particular domain and an authoritative claim to policy-relevant knowledge in that domain or issue area [17]”, may play an important role in nudging the public to take effective and efficient actions without legislation [18]. This epistemic community may also help citizens act according to expectations independently and voluntarily and may reduce the needs of aggressive interventions by the government. Although there is a study that suggests the efficacy of such a strategy in a specific field [19], further research is needed to elucidate the effective ways to achieve population health in disaster settings.

This study has several limitations. The first limitation is that the study relied only on participant responses to determine whether or not they “experienced COVID-19 infection”, which was the primary outcome variable. As of 1 November 2020, there was a cumulative total of 101,368 people who tested positive by PCR test according to the Japanese Ministry of Health, Labour and Welfare. It translates to only 0.1% of the entire population of Japan testing positive. In our study, 0.48% of the total valid respondents said that they had been diagnosed as having COVID-19 infection, which is about three times more than that of the Japanese ministry’s. Thus, it is highly likely that there was an upward bias in our study. For example, individuals with an infection experience could have more actively sought to participate in our study because of their increased interest in the significance and content of this online survey, causing an upward bias in participation of this type of subject. The RIETI questionnaire survey used self-reported information on their SARS-CoV-2 diagnosis at a medical facility to establish the presence or absence of SARS-CoV-2 infection experience. The study, therefore, does not include information on individuals who could have had SARS-CoV-2. These individuals might not have received the diagnosis because they were asymptomatic or only had mild infection and recovered without medical intervention. If the individuals with a diagnosis differed from asymptomatic or mild cases in their behavioral pattern or individual characteristics, such differences could have introduced a constant bias into the analysis results.

The second limitation is that the study was cross sectional. Therefore, a causal relationship cannot be determined between infection and behavior: individuals with a SARS-CoV-2 diagnosis could have been more careful in their daily lives. This possibility is supported by our result that the SARS-CoV-2 group had only a few individuals who had an exercise habit. This habit seems to increase the infection risk, as previously mentioned. Considering the likelihood of such bias, interpretation of estimates should be carefully examined (such as the average treatment effect), particularly the interpretation of the level of effect size. The RIETI questionnaire survey is a panel survey. Even if there were biases from active participation of the aforementioned type of individuals, the data might not show newly confirmed SARS-CoV-2 cases in second and later surveys conducted at our scale. Therefore, second and later surveys should also be analyzed in the same way.

Given these considerations of limitation, infection was still more strongly and negatively correlated with hand washing and mask wearing compared with other behaviors. This result is important in devising effective and sustainable infection control in the future.

## 5. Conclusions

This study analyzed correlation of behavioral factors and diagnosis of SARS-CoV-2 infection in Japan. Our findings suggested that curfews and remote working might not necessarily lead to sufficient reduction of the infection risk of the entire society, at least in Japanese society. Instead, appropriate preventive actions such as hand sanitizing and mask wearing are the first priorities. For long-term infection control, it is important to utilize efficient behavioral intervention. At the same time, it is important to avoid excessive interventions that are less effective, so that people can minimize the indirect harm and economic loss due to curfews and other restrictions.

## Figures and Tables

**Table 1:** Background of the participants. Difference between the SARS-CoV-2 group and the control group were calculated by chi-squared test.

Variables	Categories	SARS-CoV-2 Group (N = 44)		Control Group (N = 13,277)		*p*
		N	%	N	%	
Age group	18–19	2	4.5	297	2.2	<0.01
	20–29	12	27.3	1270	9.6	
	30–39	8	18.2	1479	11.1	
	40–49	10	22.7	2698	20.3	
	50–59	7	15.9	2863	21.6	
	60–69	3	6.8	3076	23.2	
	70–74	2	4.5	1594	12.0	
Gender	Female	16	36.4	6566	49.5	0.09
	Male	28	63.6	6711	50.5	
BMI	<18.5	3	6.8	1736	13.1	0.39
	18.5–25	31	70.5	9044	68.1	
	25–30	9	20.5	2097	15.8	
	≥30	1	2.3	400	3.0	
Pre-existing condition	High blood pressure	11	25.0	2179	16.4	0.13
	Lipid abnormalities	5	11.4	1242	9.4	0.65
	Diabetes	5	11.4	671	5.1	0.06
	Heart disease	5	11.4	299	2.3	<0.01
	Renal disease	1	2.3	102	0.8	0.26
	Cancer	1	2.3	201	1.5	0.68
	Lung or respiratory disease	1	2.3	299	2.3	0.99
	Other condition *	2	4.5	184	1.4	0.08
Lifestyle	Avoid poorly ventilated places	36	81.8	11,348	85.5	0.49
	Avoid places where many people gather	29	65.9	11,570	87.1	<0.01
	Avoid talking or projecting voice near someone	31	70.5	10,664	80.3	0.10
	Wear a mask	37	84.1	12,915	97.3	<0.01
	Wash hands	34	77.3	12,808	96.5	<0.01
	Disinfect hands	36	81.8	11,848	89.2	0.11
	Change clothes frequently	22	50.0	2820	21.2	<0.01
	Gargle	28	63.6	9122	68.7	0.47
	Disinfect belongings	24	54.5	3743	28.2	<0.01
	Keep distance from others when going out	30	68.2	10,937	82.4	0.01
	Refrain from visiting hospitals and clinics as much as possible	22	50.0	6572	49.5	0.59
	Try to go out as seldom as possible	30	68.2	8082	60.9	0.32
Frequency of working from home	Largely all of the time	6	13.6	900	6.8	<0.01
	Half or more of the time	5	11.4	359	2.7	
	Less than half or more of the time	3	6.8	533	4.0	
	Almost never	18	40.9	5952	44.8	

N.A.: not applicable. * Disease due to which you were prohibited by a doctor from exercising, or disease or injury due to which you have major difficulties in walking (e.g., rheumatoid arthritis and bone fracture).

**Table 2:** Comparisons of COVID-19 and control groups using two statistical methods. Multiple logistic regression (left column) and inverse-probability weighing method (IPW, right column) were conducted. IPW was controlled for age and coexistence with heart disease. Odds ratio and average treatment effects of being in the COVID-19 group are shown.

Variables	Multiple Regression				IPW			
	OR	95%CI		*p*	ATE (%change)	95%CI		*p*
Age	0.94	0.91	0.98	<0.01	N.A.			
Male gender	0.96	0.87	1.06	0.47				
BMI	0.91	0.43	1.92	0.81				
**Pre-existing condition**								
High blood pressure	2.72	0.73	10.15	0.14	195.0	−78.7	468.6	0.16
Lipid abnormalities	1.27	0.38	4.25	0.69	107.2	−118.3	332.7	0.35
Diabetes	0.89	0.13	5.86	0.90	194.8	−107.6	497.2	0.21
Heart disease	11.33	2.50	51.25	<0.01	2704.4	−918.2	6327.0	0.14
Renal disease	0.95	0.12	7.78	0.96	912.9	−964.4	2790.2	0.34
Lung or respiratory disease	1.26	0.26	5.99	0.77	−62.1	−140.8	16.5	0.12
Other condition *	6.03	1.41	25.77	0.02	152.4	−336.5	641.3	0.54
**Lifestyle**								
Avoid poorly ventilated places	2.31	0.34	15.56	0.39	443.6	−324.1	1211.2	0.26
Avoid places where many people gather	0.27	0.06	1.22	0.09	−62.2	−115.6	−8.7	0.02
Avoid talking or projecting voice near someone	1.15	0.36	3.67	0.81	−19.0	−81.0	43.1	0.55
Wear a mask	0.66	0.17	2.55	0.54	−86.4	−175.4	2.6	0.06
Wash hands	0.10	0.02	0.56	0.01	−84.8	−155.8	−13.7	0.02
Disinfect hands	1.30	0.27	6.38	0.74	−44.0	−117.8	29.9	0.24
Change clothes frequently	2.96	1.08	8.15	0.04	274.4	113.2	435.6	<0.01
Gargle	0.98	0.34	2.85	0.97	−16.3	−74.3	41.7	0.58
Disinfect belongings	3.78	1.37	10.44	0.01	100.0	55.9	144.1	<0.01
Keep distance from others when going out	0.43	0.12	1.51	0.19	−43.8	−102.4	14.8	0.14
Refrain from visiting hospitals and clinics as much as possible	0.38	0.13	1.12	0.08	2.1	−59.5	63.7	0.95
Try to go out as seldom as possible	3.20	1.03	9.88	0.04	45.2	−28.6	119.0	0.23
**Frequency of working from home**								
Largely all of the time	1 (Reference)				0 (Reference)			
Half or more of the time	2.79	0.71	10.97	0.14	15.1	−96.8	1.8	0.79
Less than half or more of the time	1.09	0.25	4.78	0.91	−67.8	−142.6	0.1	0.08
Almost never	0.62	0.20	1.97	0.42	−77.1	−143.4	−0.2	0.02

IPW: inverse probability weighting analysis, OR: odds ratio; CI: confidence interval; N.A.: not applicable, ATE: average treatment effect. * Disease due to which you were prohibited by a doctor from exercising, or disease or injury due to which you have major difficulties in walking (e.g., rheumatoid arthritis and bone fracture).

## Data Availability

Data was obtained by NTTCom Online Marketing Solutions Corporation and are not publicly available.

## Supplementary Materials

The following are available online at https://www.mdpi.com/article/10.3390/ijerph182212184/s1, Table S1: Contents of questionnaire; Table S2, Results of sensitivity analysis.
