# Supplementary material for: Behavioral Factors Associated with COVID-19 Risk: A Cross-Sectional Survey in Japan
Source: Int J Environ Res Public Health. 2021 Nov 20;18(22):12184. doi: 10.3390/ijerph182212184 (PMC8625679; doi:10.3390/ijerph182212184)
Supplement: Supplementary file 1 [file ijerph-18-12184-s001.zip › ijerph-1445403-supplementary.pdf]

Table S1. Contents of questionnaire

**I. Have you been diagnosed with COVID-19 at a medical facility?**<br>1. I have been diagnosed with COVID-19 at a medical facility and am currently under treatment.<br>2. I was diagnosed with COVID-19 at a medical facility and have already recovered.<br>3. I have not been diagnosed with COVID-19 or have not seen a doctor.
**II. Which disease(s) do you currently have? Select all that apply from the following choices.**<br>1. High blood pressure<br>2. Lipid abnormalities<br>3. Diabetes<br>4. Heart disease<br>5. Kidney disease<br>6. Cancer<br>7. Lung or respiratory disease<br>8. Disease due to which you were prohibited by a doctor from exercising, or disease or injury due to which you have major difficulties in walking (e.g., rheumatoid arthritis and bone fracture)
**III. Which of the following are you conscious about to avoid contracting COVID-19?**<br>1. I do not go to poorly ventilated places.<br>2. I do not go to places where many people gather.<br>3. I do not talk or project my voice near someone.<br>4. I wear a mask.<br>5. I wash my hands.<br>6. I disinfect my hands.<br>7. I change my clothes frequently.<br>8. I gargle.<br>9. I disinfect my belongings.<br>10. I keep distance from others when I go out.<br>11. I refrain from visiting hospitals and clinics as much as possible.<br>12. I try to go out as seldomly as possible.
**IV. Please tell me the average days you exercise in a week in number.**
**V. Please tell me the average number of hours that you exercise in a day.**<br>1. Less than 30 minutes.<br>2. 30 min or more but less than 1 hour<br>3. 1 h or more but less than 2 hours<br>4. 2 hours or more
**VI. Which of the following is your main exercise?**<br>1. Walking<br>2. Running<br>3. Weight training<br>4. Calisthenics or stretching<br>5. Other
**VII. How has the amount of exercise changed compared with the same time last year?**<br>1. Decreased greatly<br>2. Decreased slightly<br>3. No change<br>4. Increased slightly<br>5, Increased greatly
**VIII. How often have you gone out in the past one month?**<br>1. Almost daily<br>2. 4-5 days per week<br>3. 2-3 days per week<br>4. About 1 day per week<br>5. About 1 day per month<br>6. Not gone out at all
**IX. Have you worked from home in the past one month?**<br>1. Largely worked from home<br>2. Worked from home half or more of the time<br>3. Worked from home less than half of the time<br>4. Almost never

Table S2. Results of sensitivity analysis.

		Multiple regression				IPW			
		OR	95% CI		p	ATE	95% CI		p
Age		0.95	0.93	0.97	<0.01				
Gender	Male	1.01	0.94	1.09	0.83				
BMI		1.41	0.80	2.50	0.23				
Pre-existing condition	High blood pressure	1.56	0.69	3.53	0.28	206.4	-14.5	427.3	0.07
	Lipid abnormalities	1.16	0.42	3.19	0.78	54.4	-74.7	183.5	0.41
	Diabetes	2.27	0.78	6.59	0.13	85.8	-69.0	240.7	0.28
	Heart disease	3.13	0.82	11.90	0.10	859.6	-175.7	1894.9	0.10
	Other condition*	1.61	0.20	12.98	0.65	119.4	-189.3	428.1	0.45
Lifestyle	Avoid poorly ventilated places	4.31	1.65	11.28	<0.01	24.2	-19.5	67.9	0.28
	Avoid places where many people gather	0.31	0.13	0.74	0.01	-13.3	-49.2	22.7	0.47
	Avoid talking or projecting voice near someone	2.20	0.90	5.35	0.08	6.4	-33.6	46.4	0.75
	Wear a mask	1.03	0.38	2.82	0.95	-20.3	-57.0	16.5	0.28
	Wash hands	0.36	0.12	1.05	0.06	-29.5	-63.7	4.7	0.09
	Disinfect hands	0.41	0.18	0.95	0.04	-30.5	-62.8	1.9	0.07
	Change clothes frequently	2.16	0.93	5.03	0.07	248.0	100.1	395.8	<0.01
	Gargle	1.87	0.87	4.00	0.11	10.6	-30.4	51.6	0.61
	Disinfect belongings	2.05	0.91	4.62	0.08	140.8	44.2	237.4	<0.01
	Keep distance from others when going out	0.30	0.14	0.66	<0.01	-17.6	-52.8	17.6	0.33
	Refrain from visiting hospitals and clinics as much as possible	1.10	0.54	2.21	0.80	57.1	-7.1	121.2	0.08
	Try to go out as seldom as possible	1.29	0.65	2.56	0.47	0.0	-40.2	40.2	1.00
Frequency of working from home	Largely all of the time	1 (Reference)				0 (Reference)			
	Half or more of the time	2.28	0.91	5.71	0.08	106.5	-34.0	247.1	0.14
	Less than half or more of the time	1.53	0.57	4.09	0.40	15.3	-89.6	120.1	0.78
	Almost never	0.68	0.32	1.44	0.31	-49.1	-117.6	19.3	0.16

IPW: inverse probability weighting analysis, OR: odds ratio; N.A. : not applicable, ATE: average treatment effect, CI: confidence interval

* Disease due to which you were prohibited by a doctor from exercising, or disease or injury due to which you have major difficulties in walking (e.g., rheumatoid arthritis and bone fracture)
